# Third-Generation Cephalosporin Resistance in Intrinsic Colistin-Resistant Enterobacterales Isolated from Retail Meat

**DOI:** 10.3390/antibiotics10121437

**Published:** 2021-11-24

**Authors:** Justice Opare Odoi, Sayo Takayanagi, Montira Yossapol, Michiyo Sugiyama, Tetsuo Asai

**Affiliations:** 1 Department of Applied Veterinary Science, United Graduate School of Veterinary Sciences, Gifu University, Gifu 501-1193, Japan; wentworthprince@yahoo.com (J.O.O.); michon@gifu-u.ac.jp (M.S.); 2 Faculty of Applied Biological Sciences, Gifu University, 1-1 Yanagido, Gifu 501-1193, Japan; takayanagi-sayo@pref.gifu.1g.jp; 3 Bioveterinary Research Unit, Faculty of Veterinary Sciences, Mahasarakham University, Maha Sarakham 44000, Thailand; montira.y@msu.ac.th; 4 Education and Research Center for Food Animal Health, Faculty of Applied Biological Sciences, Gifu University (GeFAH), 1-1 Yanagido, Gifu 501-1193, Japan

**Keywords:** Enterobacterales, intrinsic colistin resistance, retail meat

## Abstract

Consumption of retail meat contaminated with antimicrobial-resistant (AMR) bacteria is a common route for transmitting clinically relevant resistant bacteria to humans. Here, we investigated the genotypic and phenotypic resistance profiles of intrinsic colistin-resistant (ICR) Enterobacterales isolated from retail meats. ICR Enterobacterales were isolated from 103 samples of chicken, 103 samples of pork, and 104 samples of beef purchased from retail shops in Japan, using colistin-containing media, and their antimicrobial susceptibility was examined. *Serratia* spp. (440 isolates) showed resistance to cefotaxime (19 isolates, 4.3%), tetracycline (15 isolates, 3.4%), and other antimicrobials (<1%). *Hafnia* spp. (136) showed resistance to cefotaxime (12 isolates, 8.6%), ceftazidime (four isolates, 2.9%), and tetracycline (two isolates, 1.4%). *Proteus* spp. (39) showed resistance to chloramphenicol (four isolates, 10.3%), sulfamethoxazole-trimethoprim (four isolates, 10.3%), cefotaxime (two isolates, 5.1%), kanamycin (two isolates, 5.1%), and gentamicin (one isolate, 2.6%). *Cedecea* spp. (22) were resistant to tetracycline (two isolates, 9.1%) whereas *Morganella* spp. (11) were resistant to tetracycline (four isolates, 36.4%) and chloramphenicol (one isolate, 9.2%). The resistance genes *bla*_fonA_, *bla*_ACC_, and *bla*_DHA_ were detected in cefotaxime-resistant *Serratia* spp., *Hafnia* spp., and *Morganella* spp. isolates, respectively. This emergence of antimicrobial resistance in ICR Enterobacterales may pose a public health risk.

## 1. Introduction

Antimicrobial resistance in bacteria is a major health concern for humans and food-producing animals. Resistance genes encoded in the chromosomes or plasmids that mediate antimicrobial resistance in bacteria are expressed in different forms comprising target protection, target modification, and the inhibition of intracellular antimicrobial penetration to ensure bacteria survival against antimicrobials [1]. In general, the chromosomally encoded resistance genes are vertically transferred whereas the resistance genes coded on plasmids are horizontally transferred to other bacteria, including distantly related ones [1]. An increased presence of antimicrobial-resistant (AMR) bacteria limits treatment options and efficacy. Thus, pathogenic bacteria that acquire antimicrobial resistance pose a high risk to human and animal health [2].

Colistin (CST), whose usage was discontinued due to risks of nephro- and neurotoxicity, has been reintroduced as a last resort treatment against multidrug-resistant (MDR) gram-negative bacteria (GNB) [3]. However, some Enterobacterales, namely *Serratia* spp., *Hafnia* spp., *Cedecea* spp., *Proteus* spp., *Morganii* spp., and *Providencia* spp. are intrinsically resistant to CST [3,4,5]. Intrinsic resistance is a natural resistance that bacteria exhibit to certain antimicrobial agents due to the presence of elements that occur independently of previous antimicrobial exposure and horizontal gene transfer [6]. The mechanism of intrinsic colistin resistance is mediated by the expression of the *arn*BCADTEF operon and the *eptB* gene, which add 4-amino-4-deoxy-l-arabinose and phosphoethanolamine to the lipopolysaccharides (LPS) of GNB to increase the net positive charge, thereby reducing the binding affinity of CST to LPS [3]. This renders the use of CST against GNB to be less effective.

The use of antimicrobials in food-producing animals has become widespread to prevent, control, and treat infections and to improve growth and feed efficiency [7]. Different antimicrobial agents belonging to various classes, such as tetracycline, cephalosporin, quinolones, and aminoglycoside, have been extensively used in poultry, pig, and cattle production [8]. Although most developed countries, including Japan, impose strong restrictions against the use of antimicrobials, the practice has contributed to the emergence of AMR bacteria in the food-producing animal sector [9]. Animal-based food products, especially meat, may be contaminated with AMR bacteria. The consumption of retail meat contaminated with AMR bacteria is a possible route for transmitting clinically relevant AMR bacteria to humans [10,11]. To control antimicrobial resistance, effective monitoring is an important step in identifying the sources and the potential transmission route [7]. Moreover, bacteria developing resistance to potent and relatively novel antimicrobial agents can be tracked and the underlying mechanism can be examined. Several studies have reported that Enterobacterales isolated from retail meat products are resistant to third-generation cephalosporins, carbapenems, and other classes of antimicrobial agents [12,13,14,15]. Enterobacterales can rapidly develop resistance via the transfer of AMR plasmids [16,17,18]. Intrinsic colistin-resistant (ICR) Enterobacterales [3,4,5] may acquire resistance to different antimicrobial agents from other bacteria, such as *Escherichia coli*, via mobile genetic elements.

Our previous study showed that retail meats (chicken, beef, and pork) purchased in Japan were contaminated with AMR bacteria, including ICR Enterobacterales [19]. Investigating the genotypic and phenotypic resistance profiles of ICR Enterobacterales isolated from retail meat is useful for assessing its safety for human consumption. Therefore, in the present study, we aimed to determine the antibiogram and resistance genes of ICR Enterobacterales.

## 2. Results

### 2.1. Isolated ICR Enterobacterales

In Table 1, we present the results of various Enterobacterales isolated from retail chicken (*n* = 103), pork (*n* = 103), and beef (*n* = 104). Of all the retail meat samples tested, 81.3% (252/310) were found positive for *Serratia* spp., *Hafnia* spp., *Proteus* spp., *Cedecea* spp., *Providencia* spp., and/or *Morganella* spp. The *Serratia* species *S. liquefaciens*, *S. marcescens*, and *S. fonticola* were frequently isolated from chicken, pork, and beef. In contrast, *S. plymuthica* was rarely isolated from chicken and pork. *Hafnia alvei* was another notable bacterium isolated from chicken, pork, and beef. Furthermore, *Proteus penneri* was isolated from chicken, pork, and beef whereas other *Proteus* species, *P. hauseri*, *P. mirabilis*, and *P. vulgaris,* were isolated only from chicken and pork samples. Additionally, *Cedecea davisae* and *Morganella morganii* were isolated from the chicken, pork, and beef samples, whereas *Providencia rustigianii* was isolated only from chicken.

### 2.2. Antimicrobial Resistance Profiles of the Isolated ICR Enterobacterales

ICR Enterobacterales with additional resistance to other antimicrobial agents were isolated from chicken, pork, and beef samples purchased from shops in Japan. In Table 2, we present the resistance profile of the ICR Enterobacterales isolated from the chicken, pork, and beef samples. All isolated ICR Enterobacterales strains were confirmed resistant to CST by the susceptibility testing. In addition, all the strains were susceptible to meropenem (MEM) and amikacin (AMK). The *S*. *liquefaciens* isolates were resistant to cefotaxime (CTX), ceftazidime (CAZ), and chloramphenicol (CHL). *S. marcescens* isolates mostly showed resistance to tetracycline (TET). However, some *S. marcescens* also showed resistance to chloramphenicol (CHL), ciprofloxacin (CIP), levofloxacin (LVF), and nalidixic acid (NAL). Additionally, *S. fonticola* isolates showed resistance to only CTX, though all *S. plymuthica* isolates were susceptible. Within the *H. alvei*, resistance to CTX, CAZ, TET, and NAL was observed and MDR *H. alvei* was also isolated. *Proteus* spp. showed resistance to different antimicrobial agents, with some isolates having MDR phenotype (Table 2).

**Figure 1:**
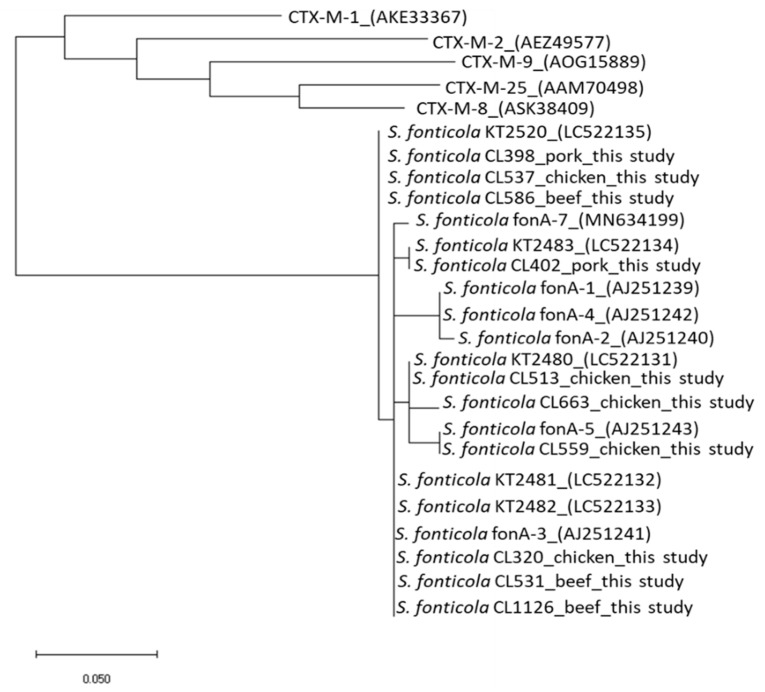
Phylogenetic comparison of the deduced amino sequences of the resistance gene *bla*fonA identified in *Serratia fonticola* isolated from chicken, pork, and beef purchased in Japan in this study with other sequences downloaded from GenBank. The relationship between *bla*fonA and CTX-M was also determined. The sources of the isolates identified in this study are indicated in the figure, and the GenBank accession numbers of the reference strains used are shown in parentheses.

Not all the third generation-resistant isolates were positive for the extended spectrum beta-lactamase (ESBL)/AmpC beta-lactamase genes tested. In contrast, the resistance gene *bla*_fonA_ was detected in 10 *S. fonticola* isolates, *bla*_ACC_ was detected in 11 *H. alvei* isolates, and *bla*_DHA_ gene was detected in one *M. morganii* isolate showing resistance to CTX (Table 3). Phylogenetic analysis showed that the *bla*_fonA_ detected in this study was homologous to other previously reported genes (Figure 1). In particular, the *bla*_fonA_ detected in CL559 was closely related to *bla*_fonA-5_, and those detected in CL320, CL531, and CL1126 were closely related to *bla*_fonA-3_. The remaining *bla*_fonA_ genes (CL398, CL402, CL513, CL537, and CL586) were closely related to other previously reported unclassified *bla*_fonA_ genes (Figure 1).

## 3. Discussion

This study demonstrated the antimicrobial susceptibilities of ICR Enterobacterales isolated from retail samples of chicken, pork, and beef meat. The isolates exhibited additional resistance to aminoglycoside (gentamicin, GEN; kanamycin, KAN); third-generation cephalosporins (CTX, CAZ); fluoroquinolone and quinolone (CIP, LVF; NAL); phenicol (CHL); sulfamethoxazole-trimethoprim, SXT; and TET. ICR Enterobacterales with additional resistance to other antimicrobial agents may further limit treatment options in case of human infections.

The presence of resistance genes (*bla*_ACC_, *bla*_DHA_, and *bla*_fonA_) in bacteria detected in this study further augments a rising concern in the medical community because these genes can lead to treatment failure by conferring resistance against broad-spectrum cephalosporins [17,20,21]. A study conducted in Paris showed that *Klebsiella pneumoniae,* possessing the *bla*_ACC_ gene and resistant to CAZ, CTX, and ceftriaxone, was responsible for nosocomial infections [22]. Additionally, *bla*_DHA_, first characterized in *Salmonella enterica* serovar Enteritidis isolated in Saudi Arabia from the stool of patients, hydrolyzed broad spectrum cephalosporin (CAZ, CTX) [23]. The gene responsible for third-generation cephalosporin resistance was found in the CTX-resistant and CAZ-resistant isolates. We detected the *bla*_ACC_ gene, which confers CTX resistance, in 11 *H. alvei* isolates. This gene is found in some Enterobacterales and has been detected in the plasmids of most *H. alvei* [20]. Although we did not investigate the localization of the *bla*_ACC_ gene, there is a possibility of gene transferability via plasmids to other Enterobacterales, taking place in the gut of humans and food-producing animals. Furthermore, the *bla*_DHA_ gene was detected in CTX-resistant *M. morganii*. The *bla*_DHA_, a plasmid-mediated AmpC-beta-lactamase gene, has been previously found in Enterobacterales [17]. In the present study, the *bla*_fonA_ gene was found in CTX-resistant *S. fonticola* isolated from retail chicken, beef, and pork. Tanimoto et al. have also identified *bla*_fonA_ in *S. fonticola* isolated from chicken [10]. The detection of *bla*_fonA_ in other meat samples, including beef and pork, in the present study indicates the extent of its spread among bacteria in food-producing animals. The transferability of *bla*_fonA_ to other bacteria requires further investigation. Resistance genes were not detected in all the isolates showing resistance to third-generation cephalosporin agents. However, *Serratia* spp., *Hafnia* spp., *Proteus* spp., and *Morganella* spp. harbor the chromosomal *ampC* gene that encodes inducible beta-lactamase, thereby conferring intrinsic resistance to aminopenicillins and narrow-spectrum cephalosporins such as cefazolin [4,5,24,25]. The isolates could harbor the genes encoding resistance; however, these were not detected due to the limitation in our methodology, such as the lack of whole genome sequencing of the isolates. 

Multi-drug resistant bacteria can withstand the effects of different antimicrobial agents [26]. In the present study, two of the *S. marcescens* isolates were MDR, and the remaining isolates were resistant to TET and CHL (Table 2). In addition, *H. alvei*, the second most frequently isolated species from retail meat in the present study, was resistant to CTX, CAZ, NAL, and TET and showed one MDR isolate (Table 2). In another study, *H. alvei* isolated from raw horsemeat was found resistant to fosfomycin but susceptible to CTX, NAL CAZ, and TET [13]. The *Proteus* spp. isolated in the present study were resistant to CTX, CAZ, GEN, KAN, and CHL. *P. mirabilis* and *P. vulgaris* isolates exhibited the MDR phenotype (Table 2). A study by Kim et al. [15] reported that *P. mirabilis* isolated from poultry in the USA was MDR (GEN-CHL-KAN). Notably, our results showed that all ICR Enterobacterales were highly susceptible to MEM and AMK. This supports that the fact that MEM- and AMK-resistant enteric bacteria are rarely detected in retail meat products [8]. Furthermore, despite most of the ICR Enterobacterales from retail meat were fully susceptible to the antimicrobials tested, the presence of additional resistance observed in some isolates suggests the emergence of ICR Enterobacterales with extra antimicrobial resistance in retail meat.

Although we used CST–deoxycholate hydrogen sulfide lactose (DHL) selective media to culture the ICR Enterobacterales in this study, future studies investigating ICR Enterobacterales should consider using a selective media supplemented with a combination of CST and a third-generation cephalosporin as this will increase the chances of isolating third-generation cephalosporin resistant strains.

As Enterobacterales contamination of retail meats is an indicator of hygiene and post-processing contamination, the results of the present study highlight a possible breakdown of hygienic handling practices at the various stages of the meat processing and distribution chain [27]. The frequently isolated *Serratia* spp. has been implicated in causing urinary tract infections and nosocomial infection in immunocompromised individuals [21,28]. *H. alvei*, the next noteworthy bacteria isolated, has been implicated in extraintestinal infection [29]. Members of the *Proteus* spp., especially *P. mirabilis*, have been frequently associated with urinary tract and wound infections [30]. Additionally, *Cedecea* spp., *Providencia* spp., and *Morganella* spp. have also been known to cause infections in immunocompromised patients [31,32,33]. The ICR Enterobacterales implicated in some human infection further demonstrate their clinical relevance. Thus, the present study emphasizes the importance of hygienic meat handling and processing to ensure the safety of retail meat for consumption. Furthermore, attention should be paid to ICR Enterobacterales as the emergence of antimicrobial resistance in this group may pose critical public health concerns.

## 4. Materials and Methods

In our previous study [19], a total of 310 meat samples, comprising 103 chicken, 103 pork, and 104 beef samples, were purchased from retail shops in Japan between May 2017 and July 2018 and isolation of ICR Enterobacterales was performed. To briefly summarize, each meat sample was gently pressed against the surface of DHL agar supplemented with 0.1 μg/mL of CST (CST–DHL medium), using sterile forceps. The preparation was aerobically incubated at 37 °C for 16–18 h. Additionally, 5 g of each meat sample was aseptically cut and cultured in 45 mL of tryptic soy broth (enrichment medium) at 37 °C overnight. The bacterial culture obtained was then streaked onto the CST–DHL medium and aerobically incubated at 37 °C for 16–18 h. A maximum of three distinct bacterial colonies were randomly chosen from each sample. A stock culture of 10% glycerol was made and stored at −80 °C for further analysis.

Isolated ICR Enterobacterales were identified using a VITEK^®^ 2 GN identification card (Sysmex BioMérieux, Tokyo, Japan), followed by antimicrobial susceptibility testing with the broth microdilution method using frozen plates (Eiken Chemical Co., Ltd., Tokyo, Japan), as previously described [19]. Antimicrobial resistance breakpoints were interpreted according to the Clinical and Laboratory Standards Institute guidelines [5]. The resistance of ICR Enterobacterales to CST was confirmed following the European Committee on Antimicrobial Susceptibility Testing resistance breakpoints [34]. Isolates resistant to three or more antimicrobial classes have been identified as MDR [21].

Multiplex PCR was performed to detect beta-lactamase genes in all third-generation cephalosporin-resistant isolates, as described previously [35]. The multiplex PCR targeted OXA-1-like broad-spectrum beta-lactamases, ESBL including variants of CTX-M, TEM, SHV, VEB, PER, and GES, AmpC beta-lactamases comprising ACC, ACT, FOX, MOX, DHA, LAT, and MIR [35]. Additionally, using the primers described previously [12], we investigated the presence of *bla*_fonA_, a minor extended-spectrum beta-lactamase gene. Both strands of the amplified DNA fragments of *bla*_fonA_ were sequenced at the Life Science Research Center of Gifu University, Japan. The resulting amino acid sequences were analyzed using the Basic Local Alignment Search Tool (National Center for Biotechnology Information, Bethesda, MD, USA). A phylogenetic tree based on the amino acid sequence of our isolates, together with other FONA and CTX-M sequences downloaded from GenBank (National Center for Biotechnology Information, https://www.ncbi.nlm.nih.gov/genbank/, accessed on 5 August 2021), was generated using MEGA 10 (https://www.megasoftware.net, accessed on 8 August 2021).

## 5. Conclusions

The ICR Enterobacterales isolated from the retail meat samples tested in this study were found to be resistant to third-generation cephalosporins and other antibiotics. Their presence suggests that retail meat has been contaminated with AMR bacteria, which can be transmitted to humans through its consumption. Thus, hygiene standards should be properly enforced at all stages of retail meat processing to prevent contamination. More attention should be paid to ICR Enterobacterales as a rise in antimicrobial resistance in these bacteria may constitute a public health risk.

## Figures and Tables

**Table 1:** Distribution of intrinsic colistin-resistant Enterobacterales isolated from retail chicken, pork, and beef purchased from shops in Japan.

Bacteria		Total Pos. Samples (%)	Total No. of Isolates	Chicken (*n* = 103)		Pork (*n* = 103)		Beef (*n* = 104)	
Genus	Species			Pos. Samples (%)	No. of Isolates	Pos. Samples (%)	No. of Isolates	Pos. Samples (%)	No. of Isolates
*Serratia*	*liquefaciens*	194 (62.6)	387	63 (61.2)	118	64 (62.1)	135	67 (64.4)	134
	*marcescens*	25 (8.1)	29	11 (10.7)	13	6 (5.8)	7	8 (7.7)	9
	*fonticola*	20 (6.5)	21	11 (10.7)	11	4 (3.8)	5	5 (4.8)	5
	*plymuthica*	2 (1)	3	1 (1)	1	1 (1)	2	0	0
	*subtotal*	202 (65.2)	440	68 (66)	143	67 (65.0)	149	67 (64.4)	148
*Hafnia*	*alvei*	89 (28.7)	136	17 (16.5)	22	40 (38.8)	62	32 (30.8)	52
*Proteus*	*penneri*	14 (4.5)	18	11 (10.7)	13	2 (1.9)	3	1 (1)	2
	*hauseri*	7 (2.3)	9	4 (3.8)	4	3 (2.9)	5	0	0
	*mirabilis*	7 (2.3)	10	6 (5.8)	9	1 (1)	1	0	0
	*vulgaris*	2 (1)	2	1 (1)	1	1 (1)	1	0	0
	*subtotal*	29 (9.4)	39	21 (20.4)	27	6 (5.8)	10	1 (1)	2
*Cedecea*	*davisae*	14 (4.5)	22	11 (10.7)	18	1 (1)	2	2 (1.9)	2
*Providencia*	*rustigianii*	12 (3.9)	12	12 (11.7)	12	0	0	0	0
*Morganella*	*morganii*	10 (3.2)	11	7 (6.8)	7	2 (1.9)	2	1 (1)	2

Abbreviation: Pos., positive; No., number.

**Table 2:** Antibiogram of the intrinsic colistin-resistant Enterobacterales found in the retail chicken, pork, and beef samples tested.

Bacteria	Resistance Profile	No. of Chicken Samples (No. of Isolates)	No. of Pork Samples (No. of Isolates)	No. of Beef Samples (No. of Isolates)	Total No. of Samples (Total No. of Isolates)
*Serratia liquefaciens*	CTX	2 (3)	1 (1)	2 (2)	5 (6)
	CTX-CAZ		1 (1)		1 (1)
	CTX-CHL		1 (1)		1 (1)
	Susceptible	61 (115)	61 (132)	65 (132)	187 (379)
	Subtotal	63 (118)	64 (135)	67 (134)	199 (387)
*Serratia marcescens*	CAZ-TET-CIP-LVF	1 (1)			1 (1)
	CTX-NAL-CHL	1 (1)			1 (1)
	TET	5 (5)	4 (4)	2 (2)	11 (11)
	TET-CHL			1 (1)	1 (1)
	Susceptible	4 (6)	2 (3)	5 (6)	11 (15)
	Subtotal	11 (13)	6 (7)	8 (9)	25 (29)
*Serratia fonticola*	CTX	5 (5)	2 (2)	3 (3)	10 (10)
	Susceptible	6 (7)	2 (3)	2 (2)	10 (12)
	Subtotal	11 (12)	4 (5)	5 (5)	19 (22)
*Serratia plymuthica*	Susceptible	1 (1)	1 (2)		2 (3)
*Hafnia alvei*	CTX	1 (1)	4 (4)	1 (1)	6 (6)
	CAZ		2 (2)	2 (3)	4 (5)
	TET		1 (1)		1 (1)
	CTX-CAZ		3 (4)	1 (1)	4 (5)
	NAL		1 (1)		1 (1)
	CTX-CAZ-TET			1 (1)	1 (1)
	Susceptible	16 (21)	29 (50)	27 (46)	72 (117)
	Subtotal	17 (22)	40 (62)	32 (52)	89 (136)
*Proteus penneri*	CHL	1 (1)			1 (1)
	CTX	2 (2)			2 (2)
	Susceptible	9 (10)	2 (3)	1 (2)	12 (15)
	Subtotal	11 (13)	2 (3)	1 (2)	14 (18)
*Proteus mirabilis*	CHL	1 (1)			1 (1)
	SXT	1 (2)			1 (2)
	KAN-CHL-SXT		1 (1)		1 (1)
	Susceptible	4 (6)	0	0	4 (6)
	Subtotal	6 (9)	1 (1)	0	7 (10)
*Proteus vulgaris*	GEN-KAN-CHL-SXT		1 (1)		1 (1)
	Susceptible	1 (1)	0		1 (1)
	Subtotal	1 (1)	1 (1)		2 (2)
*Proteus hauseri*	Susceptible	4 (4)	3 (5)		7 (9)
*Cedecea davisae*	TET	1 (2)			1 (2)
	Susceptible	10 (16)	1 (2)	2 (2)	13 (20)
	Subtotal	11 (18)	1 (2)	2 (2)	14 (22)
*Morganella morganii*	CHL	1 (1)			1 (1)
	CTX	1 (1)			1 (1)
	TET	2 (2)	2 (2)		4 (4)
	Susceptible	3 (3)	0	1 (2)	4 (5)
	Subtotal	7 (7)	2 (2)	1 (2)	10 (11)
*Providencia rustigianii*	Susceptible	12 (12)	0	0	12 (12)
Total		96 (229)	89 (225)	67 (206)	252 (659)

Abbreviations: AMK, amikacin; CAZ, ceftazidime; CHL, chloramphenicol; CIP, ciprofloxacin; CTX, cefotaxime; GEN, gentamicin; KAN, kanamycin; LVF, levofloxacin; MEM, meropenem; NAL, nalidixic acid; SXT, sulfamethoxazole-trimethoprim; TET, tetracycline.

**Table 3:** Resistance profiles and associated resistance genes identified in third-generation cephalosporin-resistant bacteria isolated from retail chicken, pork, and beef purchased in Japan.

Bacteria	Strain No.	Source	Resistance Profile	Beta-Lactamase Gene Detected	
				ESBL	AmpC
*S. fonticola*	CL-320	Chicken	CTX	*bla*_fonA_	
*S. fonticola*	CL-398	Pork	CTX	*bla*_fonA_	
*S. fonticola*	CL-402	Pork	CTX	*bla*_fonA_	
*S. fonticola*	CL-513	Chicken	CTX	*bla*_fonA_	
*S. fonticola*	CL-531	Beef	CTX	*bla*_fonA_	
*S. fonticola*	CL-537	Chicken	CTX	*bla*_fonA_	
*S. fonticola*	CL-559	Chicken	CTX	*bla*_fonA_	
*S. fonticola*	CL-586	Beef	CTX	*bla*_fonA_	
*S. fonticola*	CL-663	Chicken	CTX	*bla*_fonA_	
*S. fonticola*	CL-1126	Beef	CTX	*bla*_fonA_	
*H. alvei*	CL-41	Beef	CTX		*bla*_ACC_
*H. alvei*	CL-177	Beef	CTX-TET		*bla*_ACC_
*H. alvei*	CL-208	Pork	CTX-CAZ		*bla*_ACC_
*H. alvei*	CL-209	Pork	CTX-CAZ		*bla*_ACC_
*H. alvei*	CL-215	Beef	CTX-CAZ		*bla*_ACC_
*H. alvei*	CL-284	Pork	CTX		*bla*_ACC_
*H. alvei*	CL-338	Chicken	CTX		*bla*_ACC_
*H. alvei*	CL-607	Pork	CTX-CAZ		*bla*_ACC_
*H. alvei*	CL-698	Pork	CTX		*bla*_ACC_
*H. alvei*	CL-774	Pork	CTX-CAZ		*bla*_ACC_
*H. alvei*	CL-954	Pork	CTX		*bla*_ACC_
*M. morganii*	CL-277	Chicken	CTX		*bla*_DHA_

Abbreviations: CAZ, ceftazidime; CTX, cefotaxime; ESBL, extended spectrum beta-lactamase; TET, tetracycline; *H. alvei*, *Hafnia alvei*; *M. morganii, Morganella morganii*; *S. fonticola*, *Serratia fonticola.*

## Data Availability

The data presented in this study are available on request from the corresponding author.
